# Supplementary material for: Impact of Gender and Age on Claim Rates of Dread Disease and Cancer Insurance Policies in Taiwan
Source: Int J Environ Res Public Health. 2021 Dec 25;19(1):216. doi: 10.3390/ijerph19010216 (PMC8750668; doi:10.3390/ijerph19010216)
Supplement: Supplementary file 1 [file ijerph-19-00216-s001.zip › ijerph-1488686-supplementary.pdf]

Supplement Table S1. Results (adjusted odds ratio) of logistic regression for dread disease insurance.

Dependent variable= Claim occurrence	Total (N_{DT}=90,901)	Male (N_{DM}=44,861)	Female (N_{DF}=45,992)
Male	1.089		
Age (Ref=30-39)			
<20	0.188	1.414	0.086***
20-29	0.639***	3.058	0.424
40-49	5.445	38.628***	2.637***
50-59	9.633***	71.509***	4.336***
≥60	13.729***	92.720***	7.577***
Big-three insurers	0.703	0.564	0.861
Channel (Ref=Direct writer system)			
Agent system	1.076	1.770	0.753
Broker system	2.097***	4.022***	1.278
Direct response system	12.637**		19.248***
Waiting period (Ref=60 days)			
30 days	0.824	0.337*	2.266
90 days	0.289*	0.090**	1.033
Ln (Insured amount)	0.810	0.613***	1.101
Policy year (ref. 2012)			
2013	1.048	0.642	1.835*
2014	1.328	0.961	2.057**
2015	0.711	0.476**	1.197
Main contract	1.994**	3.398***	1.183
Constant	0.008***	0.076	0.000***
Pseudo R^2	0.1209	0.1671	0.1080
LR chi2	267.15	187.45	117.43
P > chi2	0.0000	0.0000	0.0000
Log likelihood	-970.929	-467.136	-484.766

Note: * $p < 0.10$, ** $p < 0.05$, *** $p < 0.01$. Stata deleted the variable of the Direct response system due to collinearity when it ran the regression of male data, thus 48 male observations were not included.

Supplement Table S2. Results (adjusted odds ratio) of logistic regression for dread disease insurance (dependent variable = Cancer claim occurrence).

Dependent variable=<br>Cancer claim occurrence	Total<br>(N_{DT}=90,901)	Male<br>(N_{DM}=44,861)	Female<br>(N_{DF}=45,992)
Male	0.823		
Age (Ref=30-39)			
<20	0.142***	0.938	0.07**
20-29	0.372	2.994	0.13*
40-49	4.456***	19.012***	2.943***
50-59	7.334***	43.969***	3.522***
≥60	12.103***	60.033***	7.844***
Big-three insurers	0.558*	0.218***	0.969
Channel (Ref=Direct writer system)			
Agent system	1.163	3.013*	0.712
Broker system	1.59	3.349*	1.11
Direct response system	16.342***		28.309***
Waiting period (Ref=60 days)			
30 days	0.635	0.203**	1.883
90 days	0.254*	0.042**	1.159
Ln (Insured amount)	0.944	0.74	1.202
Policy year (ref. 2012)			
2013	0.912	0.563	1.303
2014	1.923**	1.582	2.276**
2015	0.934	0.604	1.337
Main contract	1.817	3.568*	1.204
Constant	0.002**	0.011	0***
Pseudo R^2	0.1273	0.1867	0.1209
LR chi2	209.33	137.94	109.25
P > chi2	0.0000	0.0000	0.0000
Log likelihood	-717.207	-300.481	-397.197

Note: * $p < 0.10$, ** $p < 0.05$, *** $p < 0.01$. Stata deleted the variable of the Direct response system due to collinearity when it ran the regression of male data, thus 48 male observations were not included.

Supplement Table S3. Results (adjusted odds ratio) of logistic regression for dread disease insurance (dependent variable = Non-cancer claim occurrence).

Dependent variable= Non-cancer claim occurrence	Total (N_{DT}=90,831)	Male (N_{DM}=44,861)	Female (N_{DF}=39,512)
Male	2.212**		
Age (Ref=30-39)			
<20	0.436	0.296	0.16
20-29	2.174		1.743
40-49	10.534***	10.26***	0.751
50-59	23.18***	15.651***	8.89***
≥60	20.198***		5.42
Big-three insurers	1.084		0.573
Channel (Ref=Direct writer system)			
Agent system	0.941	1.424	0.882
Broker system	3.222**	5.116***	2.087
Direct response system			
Waiting period (Ref=60 days)			
30 days	4.637	4.423	4.012
90 days	1.363		
Ln (Insured amount)	0.586**	0.501**	0.748
Policy year (ref. 2012)			
2013	1.272	0.755	4.419*
2014	0.415*	0.335*	1.001
2015	0.369*	0.354*	0.53
Main contract	2.651**	4.534**	1.278
Constant	0.007	0.111	0.002
Pseudo R^2	0.1436	0.1591	0.1310
LR chi2	106.89	75.83	33.29
P > chi2	0.0000	0.0000	0.0026
Log likelihood	-318.734	-200.369	-110.424

Note: * $p < 0.10$, ** $p < 0.05$, *** $p < 0.01$. Stata deleted the variable of the Direct response system due to collinearity when running the regression, thus 70 observations were not included. Stata deleted the variables of age 20-29, age ≥60, Direct response system, and Waiting period of 90 days due to collinearity when it ran the regression, thus 5,397 male observations were not included. Stata deleted the variables of Direct response system and Waiting period of 90 days due to collinearity when it ran the regression, thus 937 female observations were not included.

Supplement Table S4. Results (adjusted odds ratio) of logistic regression for cancer insurance.

Dependent variable= Claim occurrence	Total (N_{CT}=235,699)	Male (N_{CM}=122,465)	Female (N_{CF}=98,304)
Male	0.716***		
Age (Ref=30-39)			
<20	0.16***	0.705	
20-29	0.251***	0.924	0.079**
40-49	2.927***	4.838***	2.505***
50-59	7.547***	14.586***	5.805***
≥60	12.936***	34.229***	7.596***
Big-three insurers	0.656***	0.701*	0.634***
Channel (Ref=Direct writer system)			
Agent system	0.962	0.86	1.042
Broker system	0.686**	0.828	0.581**
Direct response system			
Waiting period (Ref=60 days)			
30 days			
90 days			
Ln(Insured amount)	1.033	0.984	1.079
Policy year (ref. 2012)			
2013	1.043	1.098	1.004
2014	1.06	1.133	1.010
2015	1.005	0.956	1.046
Main contract	1.303	0.831	2.305*
Constant	0.001***	0.001***	0.000***
Pseudo R^2	0.079	0.0931	0.0538
LR chi2	742.47	395.12	270.12
P > chi2	0.0000	0.0000	0.0000
Log likelihood	-4325.4522	-1923.6253	-2377.5382

Note: * $p < 0.10$, ** $p < 0.05$, *** $p < 0.01$. In the total (male) insureds, for collinearity, Stata deleted the variables of the Direct response system and waiting period when it ran the regression, thus 48 (24) observations were not included. In the female insureds, for collinearity, Stata deleted the variables of Age <20, Direct response system and waiting period when it ran the regression, thus 14,935 observations were not included.
